# Atrial Fibrillation among Patients Admitted to the Department of Internal Medicine in a Tertiary Care Centre: A Descriptive Cross-sectional Study

**DOI:** 10.31729/jnma.7858

**Published:** 2022-09-30

**Authors:** Surendra Prasad Shah, Ram Pratap Sah, Sagar Panthi, Rakesh Kumar Shah, Rochana Acharya, Durga Neupane, Reecha Puri, Sulaksha Poudel, Lila Bahadur Basnet

**Affiliations:** 1 Department of Internal Medicine, Nobel Medical College Teaching Hospital, Biratnagar, Morang, Nepal; 2 B. P. Koirala Institute of Health Sciences, Dharan, Sunsari, Nepal; 3 Manmohan Memorial Institute of Health Sciences, Maharajgunj, Kathmandu, Nepal; 4 Curative Service Division, Department of Health Services, Teku, Kathmandu, Nepal

**Keywords:** *atrial fibrillation*, *heart valve disease*, *hypertension*, *rheumatic heart disease*

## Abstract

**Introduction:**

Atrial fibrillation is one of the commonest arrhythmias with an overall prevalence estimated to be 0.4-1% in the general population. The objective of this study was to find out the prevalence of atrial fibrillation among patients admitted to the Department of Internal Medicine in a tertiary care centre.

**Methods:**

A descriptive cross-sectional study was conducted among patients admitted to the Department of Internal Medicine in a tertiary care centre from 01 March 2021 to 01 March 2022. Ethical approval was obtained from the Institutional Review Committee (Reference number: IRC- 478/2021). Convenience sampling method was used. Data were collected from the hospital records using a semi-structured study proforma including demography, clinical presentation, laboratory investigations, electrocardiogram, 2-dimension echocardiography, and CHA_2_DS_2-_ VASc score. Point estimate and 95% Confidence Interval were calculated.

**Results:**

Among 27,980 patients, atrial fibrillation was found in 185 (0.66%) (0.58-0.77, 95% Confidence Interval). Among them 66 (35.67%) were in the age group of 61-70 years and 97 (52.43%) were females. Dyspnea was present in 149 (80.54%), palpitation in 137 (74.05%) and pedal edema in 117 (63.27%). Valvular atrial fibrillation was seen in 101 (54.59%) and non-valvular atrial fibrillation was seen in 84 (45.41%) patients.

**Conclusions:**

The prevalence of atrial fibrillation was found to be similar when compared to other studies conducted in similar settings.

## INTRODUCTION

Atrial fibrillation (AF) is one of the most common arrhythmias with significant morbidity and mortality, tied closely with growing age.^1-3^ The overall prevalence of AF in the general population is estimated to be 0.4-1%.^2^ Various risk factors like age, hypertension, heart failure, coronary artery disease, etc. may impact the triggers for AF, which commonly arise in the pulmonary veins or the substrate for maintenance of AF.^2,3^

The prevalence of valvular heart diseases is around 30% among the patients with AF.^4^ The size and volume of the left atrium is an important factor of AF, particularly with rheumatic heart disease.^5^ Several diseases are associated with left atrial dysfunction, and it is essential to document echocardiographic findings in patients with AF.^6^

The objective of this study was to find out the prevalence of atrial fibrillation among patients admitted to the Department of Internal Medicine in a tertiary care centre.

## METHODS

A descriptive cross-sectional study was conducted among patients admitted to the Department of Internal Medicine at Nobel Medical College Teaching Hospital, Biratnagar, Nepal from 01 March 2021 to 01 March 2022. Ethical approval was obtained from the Institutional Review Committee (Reference number: IRC-NMCTH 478/2021). Patients above 18 years of age with clinically and electrocardiographically proven AF admitted were included. Informed consent was taken from the study participants. Hemodynamically unstable patients were excluded from the study. Convenience sampling method was used. The sample size was calculated using the following formula:


$$\begin{array}{ll} \text{n} & ={\text{Z}}^{2}\times \frac{\text{p}\times \text{q}}{{\text{e}}^{\text{2}}}\\ & =1.96^{2}\times \frac{0.5\times 0.5}{0.01^{2}}\\ & =9604 \end{array}$$

Where,

n= minimum required sample size
Z= 1.96 at 95% Confidence Interval (CI)
p= prevalence taken as 50% for maximum sample size calculation
q= 1-p
e= margin of error, 1%

Since convenience sampling was used in this study, the sample size was doubled and a sample size of 19,208 was calculated. After adding 10% non response rate, the required sample size was 21,343. However, 27,980 admitted cases within the study time frame were taken for the study.

Demographic information of the patients was obtained from their medical records which included the following information of the patients: clinical presentation, past medical history, comorbid conditions, medication history, personal history of smoking and alcohol intake, general physical (vitals) and systemic examination findings, laboratory investigations (complete blood count, lipid profile, liver function test, thyroid function for high risk), CHA_2_DS_2_-VASc score, 12 lead Electrocardiogram (ECG) and Transthoracic Echocardiography (TTE). Diagnosis of AF was done by absent P waves, fibrillatory waves, the irregularly irregular ventricular rate on ECG were taken as the evidence for AF.^2,4^ All patients were analyzed with 2D echocardiography, M-MODE, and color doppler to find out the coronary heart diseases if structural heart diseases like congenital heart diseases, hypertensive heart disease, and dilated cardiomyopathies, hypertrophic cardiomyopathy. TTE assessment also included the search for the presence of a left atrial clot.

Left atrial (LA) size was measured in parasternal long-axis view in all patients. Since some patients of AF do not have evidence of left heart disease and AF in them may be arising from right atrial (RA) anomaly, we also measured the sum of LA and RA size in four-chamber view. LA and RA size was taken as the largest diameter in four-chamber view.

Data were collected using a pre-designed semistructured proforma and analyzed using Microsoft Excel 2016 and IBM SPSS Statistics version 21.0. Point estimate and 95% CI were calculated.

## RESULTS

Out of 27,980 admitted patients, AF was found in 185 (0.66%) (0.58-0.77, 95% CI). The mean age of the patients was 57.91 ±6.47 years with 66 (35.67%) patients in the age group 61-70 years. A total of 97 (52.43%) patients were females. The most common presenting complaints were dyspnea seen in 149 (80.54%), palpitation in 137 (74.05%), pedal edema in 117 (63.24%), cough in 85 (45.95%) and chest pain in 77 (41.62%). Tachycardia was seen in 105 (56.76%) patients while pulse deficit of over 20 mm Hg was observed in 58 (31.35%) patients.

Valvular AF was seen in 101 (54.59%) patients and non-valvular AF was seen in 84 (45.41%). Among valvular AF, rheumatic heart disease (RHD) was found in 94 (50.81%) and among non-valvular AF hypertension was found in 24 (12.97%). A total of 31 (16.76%) patients had hypertension with ischemic heart disease (IHD) among non-valvular cases. Chronic obstructive pulmonary disease (COPD) was seen in 15 (8.11%), cardiomyopathy in 12 (6.49%), lone AF in 7 (3.78%) and thyrotoxicosis in 2 (1.08%) patients (Table 1).

**Table 1:** Coexisting pathologies observed in patients with AF (n= 185).

Etiology	n (%)
RHD	94 (50.81)
Hypertension	24 (12.97)
IHD + hypertension	31 (16.76)
COPD	15 (8.11)
Cardiomyopathy	12 (6.49)
None	7 (3.78)
Thyrotoxicosis	2 (1.08)

**Figure 1:**
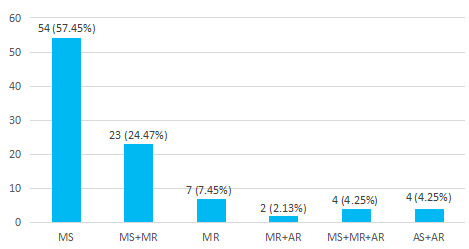
Distribution based on valvular pathology of RHD cases (n= 94).

Most common valvular pathology seen in RHD cases was isolated mitral stenosis (MS) found in 54 (57.44%) followed by MS with mitral regurgitation (MR) in 23 (24.47%). Isolated MR was seen in 7 (7.45%) cases while multiple valvular involvements were seen in 10 (10.64%) cases (Figure 1).

On ECG examination, P waves were absent in 185 (100%) of the cases. Left and right ventricular hypertrophy were seen in 32 (17.29%) and 12 (6.49%) cases respectively. Evidence of IHD was seen in 27 (14.59%) cases and left bundle branch block (LBBB) in 4 (2.16%) cases. On 2-D echocardiography, ejection fraction (EF) below 50% was observed in 110 (59.46%) cases. Left ventricle hypertrophy (LVH) was found in 46 (24.86%) cases while diastolic dysfunction (DD) in 27 (14.59%) cases. Regional wall motion abnormalities (RWMA) were observed in 19 (10.27%) cases (Table 2).

**Table 2:** Distribution based on echocardiographic changes (n= 185).

2-D echo changes	n (%)
Ejection fraction <50%	110 (59.46)
Left ventricle hypertrophy	46 (24.86)
Diastolic dysfunction	27 (14.59)
Right wall motion abnormalities	19 (10.27)

The mean left atrial size of patients with atrial fibrillation was 4.12±0.46 cm ranging from 3-5.6 cm. The majority of the cases 105 (56.76%) had left atrial size between 4-5 cm, while 38 (20.54%) cases had left atrial size <3.7 cm. The mean left atrial size of patients with rheumatic heart disease was 4.34±0.20 cm and that of non-rheumatic heart disease was 3.9±0.53 cm.

**Figure 2:**
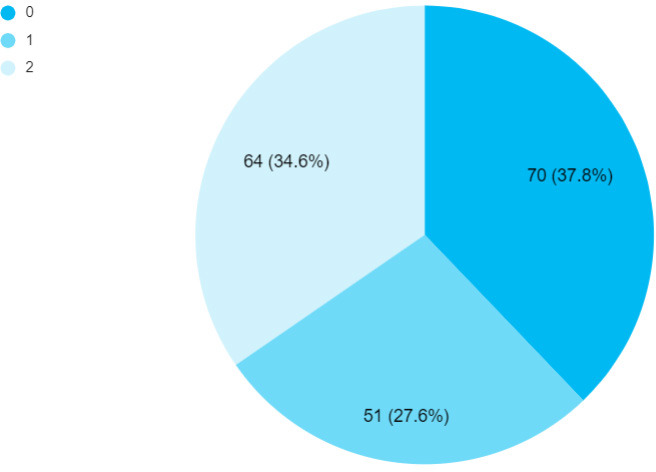
Distribution based on CHA_2_DS_2_-VASc score (n= 185).

Likewise, 144 (77.84%) developed congestive cardiac failure following AF, 15 (8.11%) had cor-pulmonale, 3 (1.62%) had superior mesenteric artery ischemia, 8 (4.32%) had ischemic stroke and 15 (8.11%) patients were seen without having any complications. Similarly, a CHA_2_DS_2_-VASc score of 0 was seen in 70 (37.84%) cases, a score of 1 in 51 (27.57%) and a score of 2 or more was seen 64 (34.59%) cases. The thromboembolic event in the form of stroke and superior mesenteric artery ischemia was seen in 11 (5.95%) cases where all of them had CHA_2_DS_2_-VASc score of 2. Mortality was recorded in 4 (2.16%) cases (Figure 2).

## DISCUSSION

The prevalence of AF among the patients admitted to the Department of Internal Medicine at our institute was 0.66%. This finding is quite less as compared to the findings from a study done in the emergency setting in a tertiary cardiac centre in Central Nepal (13.8%).^7^ Likewise, it is also lesser as compared to the studies done among the patients in the primary care setup in Brazil (1.8%) and in rural Western India (1.6%).^8,9^ On the contrary, the prevalence is higher as compared to the findings from a village setting in India (0.1%).^10^ Interestingly, the finding in our study seems in accordance with the overall prevalence of AF estimated in the general population i.e., 0.4-1%.^2^ However, the prevalence of AF among patients with RHD seems extremely high as suggested by some studies done in Nepal (36.3% and 70%) and a meta-analysis (32.8%).^7,11,12^ These variations in the prevalence of AF could be due to the differences in the sample sizes and the different settings in which the studies were conducted.

It has been seen that the incidence of AF ranges between 2-3 new cases for every 1000 people every year for 55-64 years' age group to nearly 35 new cases for every 1000 people per year for 85-94 years' age group.^13^ Its incidence is nearly 20 times in the age groups ≥80 years as compared to ≤40 years' age group and this seems to increase exponentially with the age.^2,3,13^ The mean age of patients with AF in our study was 57.91±6.47 years with maximum patients from 61-70 years of age. This corroborates with the other studies done in different settings in Nepal and India where the mean age of presentation of patients with AF was nearly in their early and late 50s.^7,9,14-18^ Meanwhile, a study from a tertiary care super-speciality referral centre in central Nepal reported a much lower mean age for AF (42.40±20.48 years).^19^ However, the mean age for AF is found to be lesser in patients with RHD while it is comparatively higher among patients with Coronary Artery Disease (CAD).^20,21^

Our study showed female dominance among patients with AF which is similar to the findings from other studies done in Nepal.^7,19,22^ Likewise, AF among RHD patients is also found to be more common among females as shown by the two studies done among RHD patients in Nepal.^11,20^ But, contrary to our findings, various studies from India and some global studies suggest AF is more common among males as compared to females.^2,3,9,10,14,23^ However, an older Framingham study among the patients with chronic AF suggested no significant differences in the incidence of AF among both the sexes.^24^

In our study, dyspnoea, palpitation, and pedal oedema were the most common presenting complaints in patients with AF. Similar findings were noted in other studies in Nepal and India.^7,15,17,25^ On the other hand, the studies from Nepal and India also reported palpitation as the most common presenting complaint with AF.^16,19^ Despite the order of presentation, the given studies recognised dyspnoea and palpitation as the most common presentations associated with AF.

In our study, valvular causes for AF comprised most of the cases. Few studies done in Nepal and a metaanalysis reported valvular causes being the dominant causes for AF.^12,19^ While other studies in Nepal reported non-valvular causes being its dominant cause.^22,25^ In our study, RHD and hypertension were respectively the leading valvular and non-valvular etiologies for AF which is in congruence with the findings of the studies done in other parts of the world.^14,15,26,27^ Likewise, in our study, among the valvular causes, mitral stenosis was more prevalent among patients with AF as compared to mitral regurgitation which is supported by two other studies done in Nepal.^11,20^ Left atrial volume is considered a risk marker for incidental AF with a risk of 43% rise in the incidence of AF when there is a 30% rise in the left atrial volume.^5,28^ Left atrial enlargement was seen in the predominant cases with AF in our study, with patients with RHD having greater enlargement in the size of the left atrium. Similar was the finding from a study done in Nepal and a meta-analysis which too showed dilated left atrium with increased mean left atrium diameter in the patients with AF.^12,19^

In our study, most of the patients had a CHA_2_DS_2_-VASc score of 0 followed by a score of 2. The score of 2 or more is always an indication to start an oral anticoagulant, and so was the case with our study where all of them with a score of 2 or more received the prophylactic oral anticoagulant. Similar was the scenario with other studies done in Nepal and India where the majority of the cases with AF received the oral anticoagulant as prophylaxis for stroke associated with AF.^14,22,25^ It is seen that the incidental risk of ischemic stroke escalates from 0.68% to 2.49% when the CHA_2_DS_2_-VASc score in patients with AF rises to 2 from 0, thus CHA_2_DS_2_-VASc score of 1 has been considered effective enough to start the novel oral anticoagulant therapy as a prophylaxis against the development of ischemic stroke in patients with AF.^29,30^

This study was a cross-sectional study, thus it is subjected to recall bias. A cohort study is thus needed to have a clearer picture of associated complications with AF. Likewise, inclusion of patients with AF from the Department of Emergency Medicine, the Intensive Care Unit, and other wards within the institute besides the Department of Internal Medicine could have helped generate a more precise prevalence of AF in an institutionalised setup.

## CONCLUSIONS

The prevalence of atrial fibrillation was similar to other studies conducted in similar settings. Awareness on the valvular heart diseases, with an attempt to secure an early diagnosis and treatment to decrease the burden of AF is recommended.
